# 

**DOI:** 10.1192/bjb.2025.10171

**Published:** 2026-06

**Authors:** Nick Neave

**Affiliations:** Hoarding Research Group, Northumbria University, UK

**Figure:**
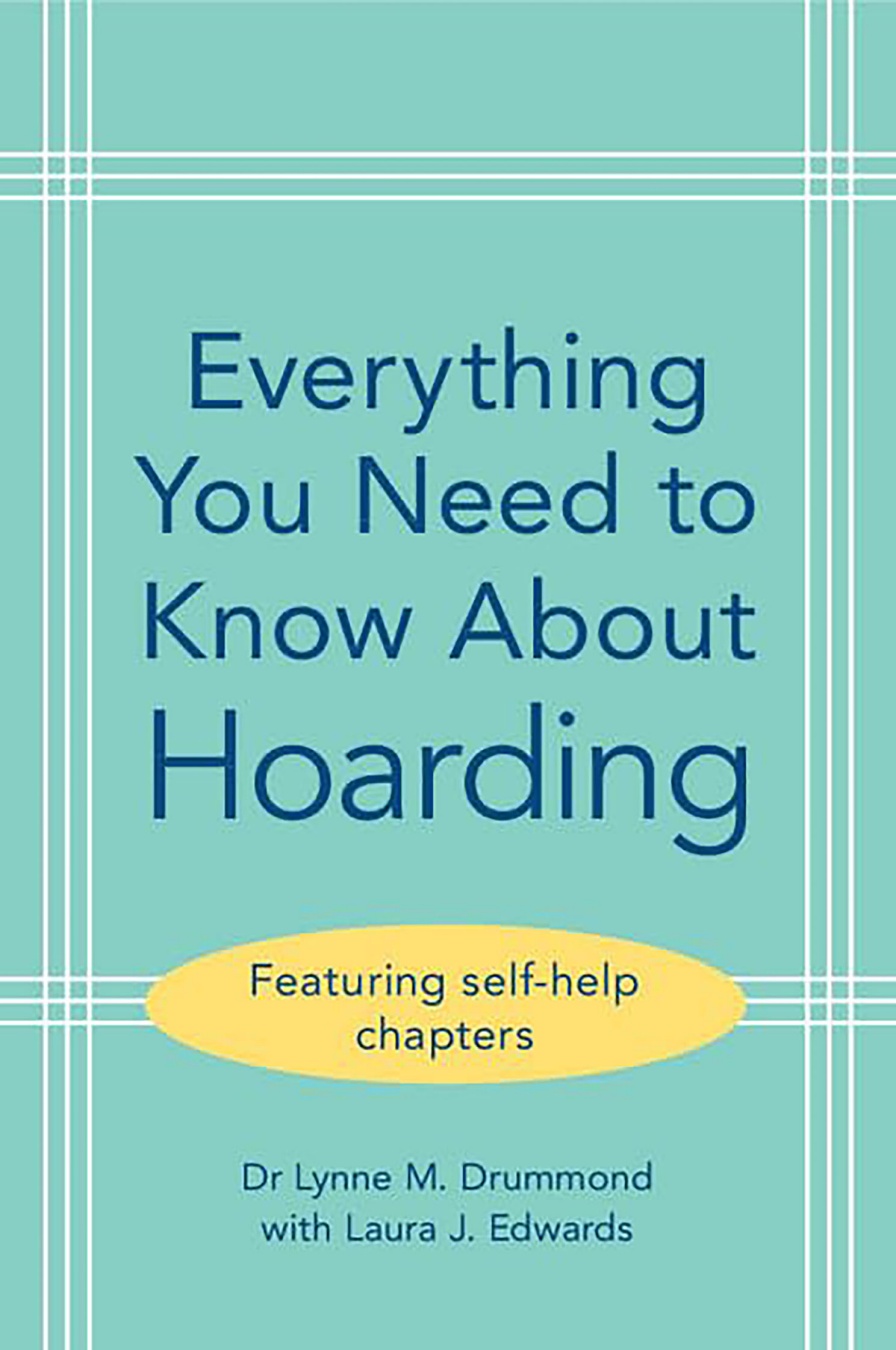

Hoarding disorder is a common, yet much misunderstood, mental health condition, often dismissed as ‘laziness’ or a ‘lifestyle choice*’*. The shame and stigma associated with hoarding (not helped by sensationalist TV programmes) means that there is a real lack of understanding about how this condition develops and manifests, though the end result can be devastating for the person who hoards and their families and the professionals who work with them. A key problem for hoarding is the lack of really effective treatment options, possibly because hoarding disorder might actually comprise different ‘types’, each with unique characteristics. Scientific papers apart, there is a real lack of information about hoarding disorder, which means that misinformation and unhelpful stereotypes abound.

This excellent book clarifies these muddy waters, providing up-to-date and accessible information about what hoarding disorder is, how it develops, its key features and how it may relate to other conditions such as obsessive–compulsive disorder, autism and attention-deficit hyperactivity disorder. The book also provides a solid review of the current treatment options and, more importantly, invaluable advice to a person with hoarding behaviours on how they can help themselves. There is also a chapter addressing how a person can provide help to a friend or relative with hoarding disorder, and this will resonate with many people currently confused and helpless in the face of seemingly bizarre behaviours by the person who hoards. A particular strength of the book is the use of vignettes and case studies drawn from the real-life experience of the first author in working with people who hoard. These offer a unique and moving insight into the condition, and how it can affect a person and their family.

This is not a textbook packed with references to research papers but can be used alongside such resources to provide a more nuanced and ‘personal’ view of this condition. It is written with compassion and insight and will be invaluable not only for students wishing to gain a deeper understanding of hoarding disorder but also for the numerous professionals who work with people who hoard and have no formal clinical/psychological training.

